# On the Association Between Mindfulness and Romantic Relationship Satisfaction: the Role of Partner Acceptance

**DOI:** 10.1007/s12671-018-0902-7

**Published:** 2018-03-13

**Authors:** Gesa Kappen, Johan C. Karremans, William J. Burk, Asuman Buyukcan-Tetik

**Affiliations:** 1 Behavioural Science Institute, Radboud University Nijmegen, Postbus 9104, 6500 HE Nijmegen, the Netherlands; 2 Faculty of Arts and Social Sciences, Sabanci University, Universite Cad. No. 27 Tuzla, 34956 Istanbul, Turkey

**Keywords:** Trait mindfulness, Partner acceptance, Relationship satisfaction, Dyadic, Couples

## Abstract

In three studies, it was investigated whether trait mindfulness is positively associated with partner acceptance, defined as the ability and willingness to accept the partner’s imperfections, and whether partner acceptance explains the association between trait mindfulness and relationship satisfaction. Trait mindfulness, partner acceptance and relationship satisfaction were assessed in two MTurk samples (*n*_1_ = 190; *n*_2_ = 140) and a sample of participants of a mindfulness-based stress reduction course (*n*_3_ = 118) and their partners (53 complete couples), using self-report measures. In all three samples, trait mindfulness was related to partner acceptance and in two out of three studies trait mindfulness was directly positively related to relationship satisfaction. Also, the results provided initial support for the mediating role of partner acceptance in the association between mindfulness and relationship satisfaction. Dyadic data further suggested that the benefits of mindfulness and partner acceptance on relationship satisfaction extend from the individual to the partner through increased partner acceptance. Together, the results provide initial support for the hypothesis that partner acceptance may be an important mechanism through which mindfulness promotes relationship satisfaction in both partners of a romantic couple.

## Introduction

An often recurring theme in both popular and scientific literature on mindfulness is that mindfulness may foster interpersonal connections by bringing open, non-judgmental attention into the interactions with people around us (e.g., Brown et al. 2007; Grayson 2004). Yet, while empirical support for the beneficial effects of mindfulness for individual well-being and health is accumulating (see e.g., Baer 2003, Brown and Ryan 2003, Keng et al. 2011), research investigating the interpersonal effects of mindfulness is still in its infancy. Initial empirical findings indicate that higher levels of trait mindfulness are generally associated with higher relationship satisfaction in romantic couples (Barnes et al. 2007; Carson et al. 2004; Kozlowski 2013; for a meta-analysis see McGill et al. 2016; Pakenham and Samios 2013; Wachs and Cordova 2007), yet, it is less clear how these benefits emerge (Karremans et al. 2017).

Cultivating the quality of a romantic relationship can be challenging, particularly because romantic partners are seldomly perfect. When asked, more than 90% of people in healthy, functioning romantic relationships report to have tried to change at least one aspect of their partner that does not align with their ideal expectations (Overall et al. 2006). The term “partner imperfection” will be used here to refer to any kind of partner behavior or trait that renders a partner less ideal in the eyes of the individual, and every now and then may trigger negative emotions such as irritation, disappointment, or anger. Partner imperfections should be understood as relatively innocuous partner behaviors that may occur in every relationship and are not inherently psychologically or physically damaging but can nevertheless lead to serious deterioration in relationship quality (Cunningham et al. 2005).

Despite the fact that partner imperfections are more rule than exception, people may be reluctant to accept that romantic partners are imperfect. Indeed, research demonstrates that partners often try to change one another into the direction of a personal romantic ideal (i.e., partner regulation; Overall et al. 2006). If successful, attempts to change a partner can contribute to both partners’ relationship satisfaction (Overall et al. 2006). However, regulation attempts often do not lead to change, and in fact may undermine relationship satisfaction for both partners (Overall et al. 2006). For example, unsuccessful partner regulation attempts increase the salience of the imperfections, and the partner who is the target of change attempt may experience a lack of appreciation. Moreover, it has been argued that the pressure of change diminishes a partner’s autonomy and triggers reactance, thereby, paradoxically, preventing change and causing distress (Cordova 2001; Jacobson et al. 2000; Sullivan and Davila 2014). Together, this suggests that often it is not the imperfection per se that negatively affects the relationship, but rather the partner’s response of attempting to change the other which may cause relationship distress (Cunningham et al. 2005; Fincham 2003, Fincham and Beach 1999).

Instead of wanting to change a partner, couples may profit from “partner acceptance,” being able to accept that the partner also has less ideal characteristics. Partner acceptance was conceptualized here as the ability and willingness to acknowledge potential imperfections of a partner without feeling the urge to change the partner (Karremans et al. 2017). While the concept has received surprisingly little explicit attention in the empirical literature on relationships, in clinical practice partner acceptance is part of various couple therapy programs such as Integrative Behavioral Couple Therapy (IBCT; Christensen et al. 1995), Acceptance and Commitment Therapy (ACT; Hayes et al. 1999), or the Couple CARE program (Rogge et al. 2002). In these programs, couples work towards realizing that some incompatibilities or imperfections are inevitable and that distress often results from one’s own emotional reactions to incompatibilities, rather than that incompatibilities are the inherently distressing factor. Some initial research findings suggest that such acceptance-based programs (in particular IBCT) benefit relationships from pre- to post-treatment with equal or even better relationship outcomes than classic, change-focused programs (e.g., traditional behavioral couple therapy; see Christensen et al. 2010; Jacobson et al. 2000). Moreover, some findings suggest that the mechanism of relationship improvement in IBCT indeed increased acceptance of negative partner behaviors (Doss et al. 2005; South et al. 2010).

Whereas some couple therapy programs, such as ACT, rely strongly on mindfulness-based techniques to increase partner acceptance, there is very little previous research that has directly examined the association between (trait) mindfulness and partner acceptance. How would mindfulness be related to partner acceptance? As noted, every now and then, partner imperfections may raise irritation, disappointment, or other negative emotions. People low in trait mindfulness have a natural tendency to immerse themselves in such emotions, which may further increase distress (e.g., Ciesla et al. 2012). Also, people low in trait mindfulness may control or suppress negative emotions, which usually increases emotional distress (Hayes et al. 1996). As a result, partners who are less tolerant to experiencing such negative emotions in their relationship—partners low in trait mindfulness—should have a stronger urge to change the partner, and be less accepting.

In contrast, people high in trait mindfulness tend to be more tolerant towards negative experiences, considering such experiences as naturally fluctuating (Creswell et al. 2007; Hayes-Skelton and Graham 2013). When encountering a partner imperfection—thus a situation in which the partner behaves in a way that does induce some irritation or anger—a person who is mindfully attending to such emotions while realizing that they are impermanent, should feel less inclined to change the partner (i.e., the “source” of the negative emotions). Put differently, an individual high in trait mindfulness should find it easier to accept that the partner is not always perfect, and that the partner sometimes behaves in ways that trigger negative emotions. Based on this argumentation, it is conceivable that trait mindfulness should be positively associated with partner acceptance.

This reasoning is in line with the rationale underlying couple programs that integrate mindfulness-based exercises to reduce avoidance of negative experiences, and as such to increase acceptability of partner behaviors in distressed couples (e.g., see Fruzzetti and Iverson 2006; South et al. 2010). Moreover, there is some empirical support for the idea that mindfulness promotes acceptance of a partner. Following a mindfulness-training program for couples, Carson et al. (2004) found that partner acceptance and relationship satisfaction increased from pre- to post-intervention. However, in addition to mindfulness exercises, the training contained various other elements of couple interventions, making it difficult to conclude whether increases in mindfulness were driving the effect (Carson et al. 2007). In a qualitative study, Pruitt and McCollum (2010) found that mindfulness meditators reported that, through their meditation, they had developed an accepting attitude towards experiences in general, as well as towards both their own and other people’s shortcomings. These preliminary findings point to the potential of mindfulness for fostering relationship quality and point to partner acceptance as a possible mechanism through which this may occur.

**Fig. 1:**
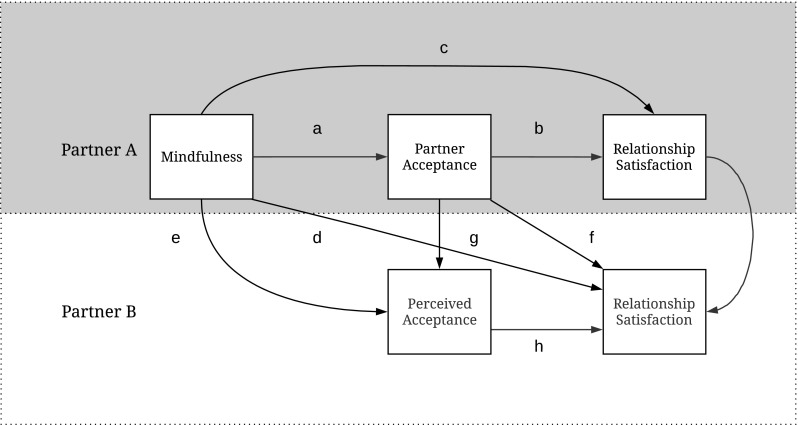
Path diagram showing conceptual model including the direct pathways tested, alphabetically named in the order in which they were tested and described in the results section. The upper part of the model (gray) was tested in Studies 1 and 2. In Study 3, the whole conceptual model was tested

Three studies were conducted to test the main prediction that, within the individual, trait mindfulness is positively associated with partner acceptance. Furthermore, partner acceptance was expected to be positively related to relationship satisfaction and based on the theoretical rationale outlined above (see Fig. 1), it was examined whether partner acceptance mediated the association between trait mindfulness and relationship satisfaction. Importantly, in Study 3, dyadic data were examined in order to answer the question whether any potential beneficial effects of trait mindfulness may extend beyond the individual to the partner’s perception of being accepted and relationship satisfaction.

## Study 1

Study 1 examined the intraindividual associations between trait mindfulness, partner acceptance and relationship satisfaction, and explored whether the link between trait mindfulness and relationship satisfaction was mediated via partner acceptance in a sample of participants who were romantically involved at the time of the study.

### Method

#### Participants

Amazon’s Mechanical Turk website was used for participant recruitment (MTurk; www.mturk.com). Of the original 224 responders, 34 participants were excluded due to incomplete or double responding, or failure to pass the control question (“If you read the instructions and if you are paying attention, please answer this question with 5 Totally Agree”). The final sample consisted of 190 participants ranging in age from 18 to 68 years (*M* = 33.66, SD *=* 10.34) with 50% being male. All participants had US citizenship and were involved in a romantic relationship with a minimum duration of 1 year, at the time of the study (1–50 years, *M =* 8.5 years, SD *=* 8.45 years). In terms of sexual orientation, the sample was 95% heterosexual (*n* = 180), 5% bisexual (*n* = 9), and 0.5% homosexual (*n* = 1). Of the complete sample, 44% (*n* = 85) were married, 3 participants did not answer this question. For correlations between demographic variables and study variables (see Table 1). Marital status had no influence on the study variables. Gender was not associated with partner acceptance, however, men were more satisfied with the relationship (*M* = 5.51, SD = .66) than women (*M* = 5.25, SD = .99; *t* (146.29) = 2.07, *p* = .04).

**Table 1:** Means, standard deviations, and correlations Study 1 and Study 2

Variable	*M*	SD	1	2	3	4
Study 1 (*N* = 190)						
1. TM	3.60	0.57				
2. PA	5.33	0.98	.35**			
3. RS	5.38	0.85	.32**	.54**		
4. Age	33.66	10.34	.22**	.10	− .00	
5. RL	8.50	8.45	.22**	.14	.08	.70**
Study 2 (*N* = 140)						
1. TM	4.38	0.42				
2. PA	5.02	1.02	.23**			
3. RS	4.04	0.82	.33**	.56**		
4. Age	35.07	11.27	.13	.11	− .20*	
5. RL			− .03	.16	− .06	.58**

*Note*. TM = Trait Mindfulness, PA = Partner Acceptance, RS = Relationship Satisfaction, RL = Relationship Length in years; ** indicates *p* < .01. M and *SD* are used to represent mean and standard deviation, respectively.

In this study, relationship length was measured using four intervals ranging from less than one year to more than ten years. The majority of participants indicated a relationship length of 1-5 years.

Data had been collected in a larger project for the purpose of which participants had been recruited with and without meditation experience based on self-selection (“Do you have experience with practicing meditation? Yes/No”). Of the final sample, 84 participants reported some experience with meditation and 106 participants did not. They were equal on all demographic variables. Regarding our variables of interest, meditators scored higher on trait mindfulness (*M* = 3.71, SD = 0.56) than non-meditators (*M* = 3.51, SD = 0.56, *t* (188) = 2.40, *p* = .02). People with meditation experience were slightly more satisfied in the relationship, *M* = 5.51, SD = .65 than people without meditation experience, *M* = 5.27, SD = .97; *t* (183.68) = 2.05, *p* = .04.

#### Procedure

Data were collected by means of online self-report questionnaires, hosted by the online survey platform Qualtrics (www.qualtrics.com). Participants first read the informed consent page, assuring anonymity and confidentiality of data processing and the voluntary nature of participation. Participants were explicitly instructed not to fill in the questionnaire together with their partners. Completion of the questionnaires took approximately 30 min. Participants completed the study in exchange for 4 US dollars. Data of all three studies were derived from larger projects. All measures used and sequences of presentation can be found in the online material.

### Measures

#### Trait Mindfulness

Trait mindfulness was assessed with the 24-item version of the Five Facet Mindfulness Questionnaire (FFMQ; Bohlmeijer et al. 2011). The FFMQ covers five facets that have been identified as the main building blocks of trait mindfulness: observing (“Generally, I pay attention to sounds, such as clocks ticking, birds chirping, or cars passing,”) describing (“I’m good at finding words to describe my feelings,”) acting with awareness (“I rush through activities without being really attentive to them,” reverse coded), accepting without judgment (“I think some of my emotions are bad or inappropriate and I shouldn’t feel them,” reverse coded), and non-reactivity (“When I have distressing thoughts or images, I don’t let myself be carried away by them”). Respondents rated different experiences on a Likert scale from 1 (never or rarely true) to 5 (often or always true). Reliability was good, *α* = .92. Responses on all items were averaged to form an overall indicator of level of trait mindfulness.

#### Partner Acceptance

Existing measures of partner acceptance did either not cover the conceptualization of the present research or had been developed for diagnostic purposes. For example, the partner responsiveness scale (Reis et al. in press) includes two items that are close to the conceptualization of the present research (“I esteem my partner, shortcomings and all” and “I value and respect the whole package that is my partner’s real self”) but the concept of responsiveness is broader, targeting receptiveness to a partner’s fundamental needs, thoughts and feelings (Reis 2014). A two-item measure of partner acceptance by Carson et al. (2004) closely matches the present conceptualization (e.g., “Considering characteristics of your partner, or your relationship, which you find difficult to deal with, over the last 2 months, how easy has it been for you to stop struggling and just allow such things to be?”) but aims at change in acceptance due to a mindfulness intervention, which makes it unsuitable for cross-sectional data collection. Furthermore, the Frequency and Acceptability of Partner Behavior Scale (Doss and Christensen 2006) measures the frequency and acceptability of a predefined set of behaviors, mainly for identifying problem areas in the relationship for clinical purposes.

To capture the idiosyncratic nature of what is considered imperfect or less than ideal in a partner, a more general measure of partner acceptance was developed. With five items, this scale measured to what extent the participant acknowledged his/her partner’s imperfections without feeling the urge to change these. Examples of positive and negative items are respectively “I can accept the less positive characteristics of my partner” and “I try to change the things which I do not like about my partner,” reverse scored. Participants rated these items on a Likert scale from 1 (totally disagree) to 7 (totally agree). To check whether this partner acceptance scale measured a construct that was separate from the general concept of relationship satisfaction, a factor analyses was conducted on all items of both scales. Initial analyses identified two factors with eigenvalues > Kaiser’s criterion of 1 for all three samples, in combination explaining 51–57% of the variance, scree plots showed inflexions pointing to two components. From subsequent factor analyses with oblique rotation, retaining 2 factors, item clustering suggested acceptance and satisfaction to be two separate components for Study 3. For Studies 1 and 2 cross loadings were found for two items of the original acceptance scale (item 1 “I appreciate my partner just the way he/she is with all his/her positive and negative aspects”; item 4 “I can accept the less pleasant characteristics of my partner”). After considering the content of the items, item 1 was removed from the scale. Notably, including the item revealed very similar results across the three studies. The scale has shown adequate reliability in pilot studies (αs > .70; Kappen 2014a, 2014b) and in the present study, *α* = .71. The full 5-item scale can be found in the Appendix. Scores on all items were averaged to create an index of partner acceptance.

#### Relationship Satisfaction

Relationship satisfaction was measured with the Relationship Assessment Scale (Hendrick et al. 1998; 7 items, e.g., “How well does your partner meet your needs?” and “In general, how satisfied are you with your relationship?”). Participants indicated how much statements were applicable to them on a 5-point Likert scale ranging from 1 (low) to 5 (high). Reliability was good, *α* = .89. Scores on all items were averaged to create an index of relationship satisfaction.

#### Data Analysis

First, the correlations between the three variables of interest were tested. Mediation was tested using the lavaan package (Rosseel 2012) in the R statistical program (R Development Core Team 2008): A simple regression model including only trait mindfulness as a predictor for relationship satisfaction was run first, followed by a mediation model including trait mindfulness as a predictor and partner acceptance as a mediator. Unstandardized path coefficients, bootstrapped standard errors and 95% confidence intervals based on bias-corrected bootstrapping are reported for all direct and indirect effects (MacKinnon et al. 2004). The proportion of the effect that was mediated was calculated by dividing the indirect effect from the mediation model by the total effect from the simple model.

### Results

Table 1 summarizes the correlations and the descriptives of the key study variables. Correlation analyses supported a positive relationship between the three variables of interest: (a) there was a positive association between levels of trait mindfulness and partner acceptance, (b) there was a positive association between partner acceptance and relationship satisfaction, and (c) trait mindfulness was positively related to relationship satisfaction.

**Fig. 2:**
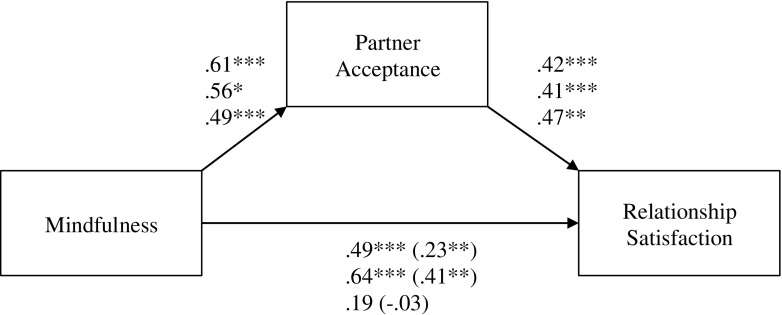
Path diagram showing the mediation mindfulness → partner acceptance → relationship satisfaction as tested in all three studies, including path coefficients from Studies 1 through 3 from top to bottom; ***p < 0.001, **p < 0.01, *p < .05

The simple regression model showed that trait mindfulness had a significant direct effect on relationship satisfaction (*b* = .49, SE = .10; 95% CIs [.30, .69]). The model including the mediator showed that participants with higher levels of trait mindfulness scored higher on partner acceptance (*b* = .61, SE = .14, 95% CIs [.31, .86]); participants scoring higher on partner acceptance were more satisfied in their relationship (*b* = .42, SE = .07, 95% CIs [.29, .56]); and partner acceptance indirectly explained the association between trait mindfulness and relationship satisfaction (*b* = .25, SE = .07, 95% CIs [.12, .42]). Including the mediator increased the amount of explained variance in satisfaction from 11 to 31%. The direct effect of mindfulness on relationship satisfaction remained statistically significant (*b* = .23, SE = .08, 95% CIs [.08, .37]), indicating that partner acceptance partially mediated the association between mindfulness and relationship satisfaction. The mediator explained 51% of the total effect (see Fig. 2 for an overview of regression coefficients for the simple mediation for studies 1 through 3).

To rule out potential third-variable explanations, the model was also tested controlling for variables that were related to the main variables (in this study, gender and meditation experience were related to relationship satisfaction). Coefficients and significant values stayed almost identical to the values attained by the uncontrolled models and are therefore not reported.

Notably, we also found support for the alternative mediation models (find an overview of alternative models across all studies in the online material). Testing every variable in the role of independent, dependent and mediating variable, we found support for the following models: mindfulness → satisfaction → acceptance; satisfaction → acceptance → mindfulness; acceptance → satisfaction → mindfulness; satisfaction → mindfulness → acceptance; acceptance → mindfulness → satisfaction. This suggests that trait mindfulness, relationship satisfaction and partner acceptance may be related in a multidirectional way. In the “Discussion”, we return to this issue.

## Study 2

The findings of Study 1 provided encouraging support in line with the main prediction that trait mindfulness is positively associated with partner acceptance within the individual. Study 2 was an attempt to replicate these findings in another sample of participants.

### Method

#### Participants

Recruitment was done in a similar way to Study 1, again using MTurk. Of the original 148 responders, eight participants were excluded due to incomplete responding or failing to pass the control question. The final sample consisted of 140 participants ranging in age from 19 to 66 years (*M* = 35.07, SD *=* 11.27) with 49% being male. All participants had US citizenship and were in a romantic relationship. Sexual orientation was not assessed. Of the whole sample, 8% had been in a relationship for less than a year, 45% had been in a relationship for 1 to 5 years, 16% had been in a relationship for 5 to 10 years, 29% had been in a relationship for ten or more years, and 2% did not provide this information. Of the complete sample, 62% (*n* = 86) were married. For correlations between demographic variables and study variables (see Table 1). Marital status and gender had no influence on the study variables. Similarly as in Study 1, participants were asked to report on their meditation experience. Those who reported meditation experience (21%) scored slightly higher on trait mindfulness (*M* = 4.53, SD = 0.39) than did non-meditators (*M* = 4.35, SD = 0.42; *t* (135) = 2.15, *p* = .03), but no effects of meditation experience on partner acceptance and relationship satisfaction were found.

#### Procedure

The procedure of Study 2 was similar to Study 1, with one exception. Instead of using the short-version of the FFMQ, trait mindfulness was now measured with the full 39-item version of the FFMQ (Baer et al. 2006; Baer et al. 2008; *α* = .86). Measures for partner acceptance (*α* = .73) and relationship satisfaction (*α* = .91) were the same as the ones in Study 1.

#### Data Analysis

Data were analyzed as in Study 1.

### Results

Correlations were similar to those found in Study 1 (see Table 1). Thus, again, we found support for our main prediction that trait mindfulness is positively association with partner acceptance. Moreover, a mediation analysis again supported the hypothesis that partner acceptance mediates the association between trait mindfulness and relationship satisfaction. The simple regression model showed that trait mindfulness had a significant direct effect on relationship satisfaction (*b* = .64, SE = .16; 95% CIs [.28, .91]). A model including the mediator showed that trait mindfulness was positively related to partner acceptance (*b* = .56, SE = .24, 95% CIs [.11, 1.05]); partner acceptance was positively related to relationship satisfaction (*b* = .41, SE = .08, 95% CIs [.24, .56]) and indirectly explained the association between trait mindfulness and relationship satisfaction (*b* = .23, SE = .11, 95% CIs [.06, .51]). Including the mediator increased the amount of explained variance in satisfaction from 11 to 35%. The mediator explained 36% of the total effect. The direct effect of mindfulness on relationship satisfaction remained statistically significant (*b* = .41, SE = .16, 95% CIs [.12, .73]), indicating that partner acceptance partially mediated the association between mindfulness and relationship satisfaction. The model was also tested controlling for variables that were related to the main variables (in this study, age was related to relationship satisfaction), which produced almost identical results.

As in Study 1, we checked alternative mediation models and found support for the two models, in which the association between trait mindfulness and partner acceptance was indirectly explained by relationship satisfaction (mindfulness → satisfaction → acceptance; acceptance → satisfaction → mindfulness). Again, this may point to multidirectional associations between the variables of interest.

## Study 3

Studies 1 and 2 provided initial support in line with the primary hypothesis: within the individual, mindfulness was positively associated with partner acceptance, and results supported that partner acceptance was in turn associated with relationship satisfaction. The goal of Study 3 was twofold. First, in addition to replicating the results of Studies 1 and 2, the aim was to examine whether trait mindfulness in one partner (partner A) affects relationship satisfaction of the other partner (partner B). Relationship quality is a result of a dyadic process in which behaviors of one partner are perceived by the other, which affect the other’s response, which again is perceived by the first partner, and so forth (e.g., Wieselquist et al. 1999). For example, literature on partner support indicates that a person’s supportive behavior benefits the partner because the partner perceives the behavior as supportive (Lemay et al. 2007). Research on the role of mindfulness in romantic relationships has paid very little attention to this notion, and there are only a few studies that have examined whether a person’s level of mindfulness is associated with the partner’s experiences in the relationship (e.g., Barnes et al. 2007; Iida and Shapiro 2017; Pakenham and Samios 2013; Karremans et al. 2017; Williams and Cano 2014). Drawing on this idea, in addition to the association between levels of trait mindfulness and partner acceptance within one partner (partner A), in Study 3, partner A’s level of mindfulness was expected to be related to partner B’s relationship satisfaction (path d in Fig. 1), through partner A’s partner acceptance and partner B’s level of perceived acceptance (a × g × h; see Fig. 1).

Second, in Study 3, these predictions were examined in a sample of participants who had at least some degree of formal training in mindfulness (i.e., had previously followed or were currently following the mindfulness-based stress reduction training). There is some debate about the validity of self-report mindfulness measures (see for example Bergomi et al. 2013; Grossman 2011). In particular, some have argued that a certain level of experience with mindfulness (training) is a prerequisite for identifying one’s own state of mindfulness, and respond to these questionnaires in a valid manner (for an extensive discussion, see Bergomi et al. 2013). Also, previous research has found differential item functioning in samples with and without mindfulness experience, indicating that responses are influenced by different biases or demands (Van Dam et al. 2009; but see Baer et al. 2010). Hence, Study 3 was conducted in a sample of mindfulness trainees, their level of trait mindfulness and partner acceptance was measured, and it was explored whether these factors in turn affected perceived acceptance and relationship satisfaction in the partner.

### Method

#### Participants

As part of a larger study, 402 accredited mindfulness trainers were approached with the request to share a survey with their trainees. Trainers’ contact information was derived from two Dutch mindfulness websites (www.instituutvoormindfulness.nl and www.vmbn.nl). Thirty-eight trainers distributed the study link among their trainees. Ten book vouchers of 50 euro each were raffled among the participants. Thirty-two participants were excluded from the analyses due to missing data (only filled in their identification codes) and 5 for not meeting the requirements for entering the study (i.e., having finished or being currently involved in a mindfulness training). The final sample consisted of 118 mindfulness trainees (i.e., partner A) and 53 matching partners (partner B), among which one same-sex couple. Among partner As, 85% (*n* = 100) completed a mindfulness training in the past and 15% (*n* = 18) were following a training at the moment when the study took place. Among partner Bs, 12% (*n* = 12) had followed a mindfulness training in the past and 2% (*n* = 2) were currently following it. Partner A and B did not differ in age (22–76 years, *M*_A_ = 48.70, SD_A_ = 11.48; *M*_B_ = 50.49, SD_B_ = 12.42, *t* (104) = − .772, *p* = .44). Couples had been together for 21.43 years on average (0–47, SD *=* 13.63). Of the complete sample, 60% (*n* = 70) were married. For correlations between demographic and study variables, see Table 2. Marital status had no influence on the study variables for neither partner As nor partner Bs. Within partner As, gender was associated with higher relationship satisfaction in men (*M* = 5.56, SD = .77) as compared to women (*M* = 5.09, SD = 1.43; *t* (58.80) = 2.15, *p* = .04). Within partner Bs, participants with meditation experience reported lower relationship satisfaction (*M* = 4.52, SD = 1.65) than participants without meditation experience (*M* = 5.71, SD = .93; t (51) = − 3.25, *p* < .01), though this might be due to the small number of partner Bs with meditation experience.

**Table 2:** Means, standard deviations, and correlations, Study 3

Variable	*M*	SD	1	2	3	4	5	6	7
1. TM_A_	5.00	0.83							
2. PA_A_	5.15	0.99	.41**						
3. RS_A_	5.18	1.34	.13	.34**					
4. PPA_A_	4.95	1.22	.27	.54**	.43**				
5. RS_B_	5.42	1.25	.05	.44**	.65**	.43**			
6. Age_A_	48.69	10.91	.35**	.07	− .09	− .03	− .18		
7. Age_B_	49.85	12.68	.29*	.08	.02	− .00	− .12	.96**	
8. RL	20.39	13.24	.10	− .03	− .10	− .04	− .16	.66**	.64**

*n*_A_ = 53; *n*_B_ = 53; *TM* trait mindfulness, *PA* partner acceptance, *PPA* perceived partner acceptance, *RS* relationship satisfaction, *RL* relationship length in years; Subscripts of A and B denote measures as assessed in partner A and B, respectively

**p < .01. *M* and SD are used to represent mean and standard deviation, respectively

#### Procedure

Partner A and B filled in similar questionnaires with the exception that partner B reported perceived partner acceptance, and not partner acceptance. Once partner A had completed the questionnaire, a link to the questionnaire was sent to partner B. In the instructions, participants were explicitly asked not to fill in the surveys together or discuss their responses with their partner before both had finished the survey.

### Measures

#### Trait Mindfulness

Trait mindfulness was assessed with the 24-item version of the FFMQ (Bohlmeijer et al. 2011; in the present study, *α* = .91). Respondents rated different experiences on a 7-point Likert scale from 1 (never or rarely true) to 7 *(*often or always true). In the present study only partner A’s trait mindfulness was analyzed for two reasons. First, as mentioned, the main goal of the present study was to replicate the findings found in Studies 1 and 2 in a sample of participants with mindfulness experience. Second, in line with the model to be tested (Fig. 1), partner Bs only provided information on their perceived partner acceptance, not their partner acceptance scores (this was also partly motivated by practical reasons, restricting the number of measures participants had to complete).

#### Partner Acceptance

Partner A (i.e., the mindfulness trainee) completed the partner acceptance scale as in Studies 1 and 2, reliability was not ideal in this sample (*α* = .62), though all items contributed to internal consistency. Partner B completed an adjusted version of this scale, using the same items but worded differently to asses perceived acceptance (e.g., “My partner tries to change the things he/she doesn’t like about me”, “My partner can accept my less pleasant characteristics”). Reliability was very good, *α* = .84.

#### Relationship Satisfaction

Relationship satisfaction was assessed with 5 items from the Investment Model scale (Rusbult, Martz, & Agnew, 1998; e.g., “I feel satisfied in our relationship”; *α* = .92). Participants indicated to what extent they agreed with the statements on a 7-point Likert scale ranging from 1 (disagree) to 7 (completely agree).

#### Data Analysis

First, the same mediation model was tested as in Studies 1 and 2, using only complete data from partner As (*n* = 105). Then, the dyadic predictions were tested (Fig. 1) including only couples for which complete data had been collected (53 partners A and 53 matching partners B) with the Lavaan package (Rosseel 2012) in the R statistical program (R Development Core Team 2008). To take interdependence between partners into account, the model adjusted for correlations between partner reports of relationship satisfaction. Unstandardized path coefficients, bootstrapped standard errors and 95% confidence intervals based on bias-corrected bootstrapping are reported for all direct and indirect effects (MacKinnon et al. 2004).

Important to note: Using only the 53 couples that provided complete data is limited, for example, because the sample may be biased (e.g., partners who did not complete the questionnaire may be less satisfied with the relationship than partner who did complete the questionnaire). The main analyses were therefore repeated using data from all couples (*n* = 118 partner As and 53 partner Bs) to estimate the parameters of the model, using full-information maximum likelihood estimation (see for details of this procedure, Allison 2012).

### Results

#### Mediation Analyses within Partner As

Table 2 summarizes the correlations among and the descriptive statistics of the key study variables. Supporting the findings from Studies 1 and 2, (a) there was a positive correlation between levels of trait mindfulness and partner acceptance, and (b) there was a positive correlation between partner acceptance and relationship satisfaction within partner A. However, although in the expected direction (*r* = .13), unlike Studies 1 and 2 trait mindfulness was not significantly correlated with self-reports of relationship satisfaction (c). The simple regression model showed that trait mindfulness did not have a significant direct effect on relationship satisfaction (*b* = .19, SE = .13; 95% CIs [− .07, .46]). A significant direct effect between a predictor and an outcome is not a necessary precondition to test mediation (Hayes 2009) as it may be the result of several mediator variables acting in opposite directions, canceling each other out. Therefore, as recommended by Hayes (2009), the product of paths (a) and (b) were estimated, using bootstrapping. In the following, such an effect will be referred to as an “indirect effect” (Hayes 2009).

A model including partner acceptance supported that mindfulness indirectly affected relationship satisfaction via partner acceptance. Specifically, trait mindfulness was positively related to partner acceptance (*b* = .49, SE = .10; 95% CIs [.31, 69]); partner acceptance was positively related to relationship satisfaction (*b* = .47, SE = .15, 95% CIs [.16, .77]); and indirectly explained the association between trait mindfulness and relationship satisfaction (*b* = .23, SE = .09, 95% CIs [.08, .44]). Including partner acceptance increased the amount of explained variance in satisfaction from 2 to 13%.Thus, although the direct effect between mindfulness and relationship satisfaction was not significant in this study, the indirect effect from mindfulness to relationship satisfaction through partner acceptance was similar to the effects obtained in Studies 1 and 2, and consistent with our main hypothesis. The model was also tested controlling for variables that were related to our main variables (in this study, gender was related to relationship satisfaction), which produced almost identical results.

As in Studies 1 and 2, we tested all alternative mediation models within partner As and found support for one alternative model in which relationship satisfaction was indirectly associated with mindfulness via partner acceptance (relationship satisfaction → partner acceptance → mindfulness). Again, this may point to multidirectional associations between the variables of interest.

#### Mediation Analyses with Dyadic Data

Correlations suggested that Mindfulness of partner A was not directly associated with partner reports (partner B) of relationship satisfaction (d), but it was significantly positively associated with perceived partner acceptance (e). Partner acceptance of partner A was positively related to relationship satisfaction of partner B (f) and to partner B’s perceived acceptance (g). Partner B’s perceived partner acceptance was positively related to partner B’s relationship satisfaction (h). Thus, in sum, these initial analyses indicate that partner A’s mindfulness again was positively associated with partner A’s partner acceptance, and also, that partner B felt more accepted to the extent that partner A was higher in mindfulness.

**Fig. 3:**
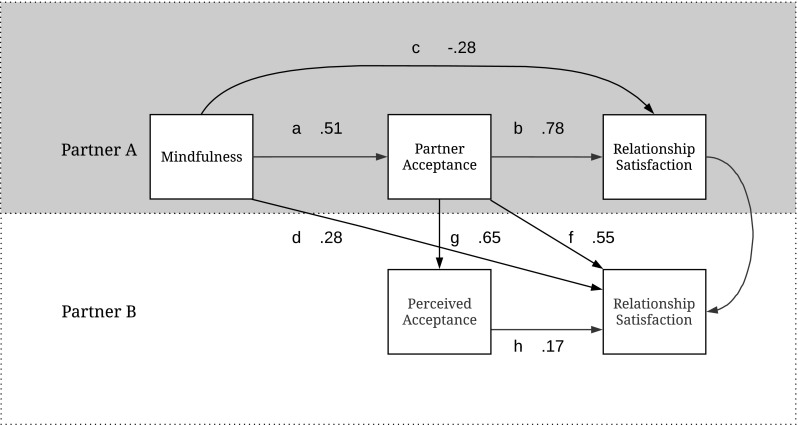
Final model, showing path coefficients of direct paths, Study 3

Next, we included the paths as depicted in Fig. 1 into an overall path analysis. Goodness of fit indices collectively indicated the estimated model did not fit the observed data *χ*^2^(1) = 2.78, *p =* .096, Comparative Fit Index (CFI) = .976, Tucker-Lewis Index (TLI) = .763, and root mean square error of approximation (RMSEA) = .183. In order to improve the model fit, the direct path from Partner A’s mindfulness to Partner B’s perceived acceptance (path e) was removed from the model. This adjustment led to an improved model fit, *χ*^2^(2) = 2.79, *p =* .248, CFI = .989, TLI = .947, RMSEA = .086. In this combined model, participants with higher levels of trait mindfulness scored higher on partner acceptance (a; *b* = .51, SE = .12, 95% CIs [.26, .73]); participants scoring higher on partner acceptance were more satisfied in their relationship (b; *b* = .78, SE = .20, 95% CIs [.36, 1.18]); and there was an indirect association between trait mindfulness and relationship satisfaction via partner acceptance (a × b; *b* = .40, SE = .14, 95% CIs [.17, .69]). However, as in the simple model and in the correlation analyses, the direct effect from partner A’s mindfulness to partner A’s own relationship satisfaction (c) was non-significant (*b* = − .28, SE = .19, 95% CIs [− .69, .04]). Is partner A’s mindfulness associated with similar outcomes in partner B? First of all, partner A’s acceptance was associated with partner B’s relationship satisfaction (f; *b* = .55, SE = .24, 95% CIs [.06 to .98]). Partner A’s partner acceptance was associated with perceived acceptance in partner B (g; *b* = .65, SE = .13, 95% CIs [.34 to .89]). Unlike the correlation analyses, in this combined model, partner B’s perceived acceptance was not associated with partner B’s relationship satisfaction, (h; *b* = .17, SE = .15, 95% CIs [− .10 to .50]). Finally, although the direct effect from partner A’s mindfulness to partner B’s relationship satisfaction (d) was not significant (*b* = − .28, SE = .19, 95% CIs [− .69, .04]), there was a significant indirect path from partner A’s mindfulness to partner A’s partner acceptance, to partner B’s relationship satisfaction, (a × f; *b* = .28, SE = .14, 95% CIs [.04, .26]). The expected complete indirect path from partner A’s mindfulness to partner B’s satisfaction via partner acceptance and perceived acceptance was not significant (b × g × h; *b* = .06, SE = .05, 95% CIs [− .02, .22]) (see Fig. 3 for an overview of path coefficients of the final model).

A model controlling for demographic variables that were significantly associated with our main variables (in this study, age of partner A was related to A’s relationship satisfaction and meditation experience of partner B was related to B’s relationship satisfaction) produced almost identical results.

As noted, we re-ran the main analyses using full-information maximum likelihood estimation to impute values for the additional 65 partner Bs with missing values on at least one of the study measures. A very similar pattern of results was obtained, with the only difference that the indirect pathway from partner A’s mindfulness to partner B’s relationship satisfaction via partner A’s partner acceptance became non-significant (a × f; *b* = .20, SE = .12, 95% CIs [− .003, .45]).

## Discussion

The present findings indicate that trait mindfulness is related to an accepting stance towards a romantic partner’s shortcomings. Data from all three studies returned a positive relationship between trait mindfulness and partner acceptance, which in turn was associated with relationship satisfaction, both in participants without and with formal mindfulness training. Study 3 provided some initial evidence that trait mindfulness of one partner can have beneficial outcomes for the other partner, in that mindfulness in partner A was associated with more partner acceptance, which was indirectly associated with higher levels of relationship satisfaction in partner B (partner A’s levels of mindfulness were not directly associated to partner B’s levels of relationship satisfaction). Partner B also felt more accepted to the extent that partner A was higher in trait mindfulness. Together the results provide initial support for the hypothesis that mindfulness is associated with partner acceptance, and that this may be an important mechanism through which mindfulness promotes relationship satisfaction.

These findings are in line with the general reasoning that approaching experiences in a mindful, non-judgmental manner may generalize to how partners cope with negative emotions that are triggered by a partner’s negative behavior or characteristic. Moreover, whereas the link between mindfulness and relationship satisfaction has been supported by several studies (for a meta-analysis see McGill et al. 2016), only little research has been conducted to examine the possible underlying mechanisms. While there certainly may be other factors at play (see for an overview Karremans et al. 2017), the present findings indicate that partner acceptance may be an important process by which mindfulness may promote relationship satisfaction. Importantly, the present data provide some evidence indicating that the effects of mindfulness in one partner operate at the level of the dyad via partner acceptance, suggesting that effects of mindfulness can extend beyond the individual (e.g., Pakenham and Samios 2013). While beneficial effects of mindfulness for the individual have been studied extensively, the present findings contribute to the emerging but young literature on the potential interpersonal benefits of mindfulness.

In the present studies, scores across all five facets of the FFMQ were used as a measure for trait mindfulness, based on the idea that the facets together represent the best index of dispositional mindfulness (e.g., Hertz et al. 2015; Jones et al. 2011). While there is some ambiguity in the literature about what is the best way to use the FFMQ, some researchers have argued that it is helpful to explore the role of the separate facets (Baer et al. 2006). However, no a priori predictions about the possible differential roles of the mindfulness facets in affecting partner acceptance had been formulated prior to conducting the present research, and the sample sizes of the studies are not ideal to include all facets separately in the models that were tested.

Relatedly, Kimmes et al. (2017) recently proposed yet another approach, which is to examine different latent profiles of the FFMQ. For example, some people may score high on observing and low on non-judgment (classified as Judgmentally Observing), others score high on awareness and high on non-judgment (classified as Non-Judgmentally Aware), and others may have high scores across the whole range of facets (High Mindfulness). They found that such classes of mindfulness as measured with the FFMQ were differentially related to anxious and avoidant attachment. In line with the notion that awareness of experiences in combination with a non-judging stance towards those experiences are both intrinsic aspects of mindfulness, it is conceivable that scoring high on *all* facets would be the profile where the level of partner acceptance is highest. Indeed, Kimmes et al. (2017), found that scoring high across all facets was most strongly related to benign attributions for partner transgressions. Nevertheless, future research should further examine the specific working mechanisms of mindfulness, and which specific (but not isolated) ingredients of mindfulness, promote partner acceptance, and relationship satisfaction.

### Strengths, Limitations, and Future Directions

There are several notable strengths of this research. The present research explicitly investigated the role of partner acceptance as a mechanism that may underlie the association between mindfulness and relationship satisfaction. For the purpose of this research, a questionnaire was composed for the assessment of partner acceptance that has strong face validity, revealed overall good reliability, and hence seems a suitable measure for further exploration of this topic. Also, the findings were consistent and fairly robust across studies (see the online materials for an overview of correlations between trait mindfulness, partner acceptance and relationship satisfaction as found in additional, unpublished projects). Moreover, Study 3 is one of the few studies to approach the interpersonal effects of mindfulness from a dyadic perspective (for notable exceptions, see Barnes et al. 2007; Iida and Shapiro 2017; Pakenham and Samios 2013; Schellekens et al. 2017; Williams and Cano 2014). Because partners in a relationship are by definition interdependent, meaning that one partner’s traits and behaviors affect the other partner’s outcomes (and vice versa), understanding the effects of mindfulness at the level of the relationship ultimately requires taking both partners into account (see Karremans et al. 2017, for an extensive discussion).

However, a number of limitations should be discussed. First, an important limitation is that the conclusions are based on participants’ self-reported level of mindfulness. For example, it has been debated whether a person can have good insight into their own (especially low) levels of mindfulness (Grossman and Van Dam 2011), and studies have shown that some items may be interpreted differently depending on participants’ meditation experience (Gu et al. 2016; Bergomi et al. 2013). Thus, in future research, it is important to examine the effects of mindfulness training and how it might affect partner acceptance and satisfaction. Second, it remains an open question as to what extent the present findings are generalizable to the general population, as there may have been sample biases. Studies 1 and 2 were based on MTurk participants, and in Study 3 (MBSR trainees) participants knew that the study concerned mindfulness and participants probably have positive attitudes towards mindfulness. Third, given the cross-sectional nature of the present findings, it remains an empirical question whether the observed association between trait mindfulness and partner acceptance translates into everyday interactions between partners. For example, earlier studies have found that levels of trait mindfulness do not necessarily predict levels of state mindfulness (Bravo et al. 2017). Future studies should address the question whether a mindful state fosters acceptance of a partner’s less-than-perfect behavior in daily life situations, using daily diaries or experience sampling methods.

Fourth, and perhaps most importantly, the present studies were correlational and cross-sectional, and thus do not provide evidence for causality. It is important for future research to examine the relationship among mindfulness, partner acceptance, and satisfaction experimentally and/or longitudinally to unravel causality and potential feedback loops between these variables. Across the studies, results did not only support a model in which partner acceptance explains the association between trait mindfulness and relationship satisfaction (this was the most consistent significant model across the three studies), but also models in which relationship satisfaction explains the relationship between trait mindfulness and partner acceptance (Studies 1 and 2), in which trait mindfulness explains the relationship between partner acceptance and relationship satisfaction (Study 1), and in which satisfaction was indirectly associated with mindfulness via partner acceptance (Study 3). Although these findings do not necessarily undermine the validity of the predicted model, they may point to the potential reciprocal nature of the associations between these variables. Aside from the proposed model, an accepting attitude towards a partner may also make it easier for people to stay mindful and satisfied, and high satisfaction may facilitate mindfulness and partner acceptance. Also at the dyadic level, partners’ levels of acceptance may mutually reinforce each other and more satisfying relationships may promote levels of mindfulness and partner acceptance in both partners. Future experimental and longitudinal studies should disentangle these various possible effects.

In addition to these limitations, the present findings present various possibilities for future avenues. An important general question is whether there are certain boundary conditions that qualify the present findings. For example, does mindfulness always promote partner acceptance? Research has shown that mindfulness may improve people’s ability to become aware of otherwise implicit processes (Brown and Ryan 2003; Carlson 2013). Mindfulness may therefore help people to recognize automatic or unconscious tendencies to justify a partner’s behavior, which can occur in the face of abuse (e.g., Rusbult and Martz 1995). An interesting question would therefore be whether mindfulness may in fact help partners to stop accepting severe negative partner behaviors and decide that ending the relationship might be the better option. Related to this issue, it is not clear whether partner acceptance always promotes relationship satisfaction. For example, accepting a partner’s shortcoming may result in continuation of the behavior, while sometimes a lack of acceptance may be required to motivate a partner to behave differently and improve the relationship (cf. Luchies et al. 2010; McNulty and Fincham 2012). Put differently, there may be a thin line between partner acceptance (which would promote relationship satisfaction) and resignation (which would hurt relationships). These questions are particularly important for the informed integration of mindfulness-based techniques into couple interventions. Whereas these techniques are already widely used in this context, scientific investigation into the effectiveness and boundary conditions of mindfulness interventions and partner acceptance is lacking. More scientific work is needed to clarify if and under which conditions mindfulness-based techniques can be used to support individual and relationship well-being in the context of romantic relationships.

The current research focused on the role of partner acceptance, but there may be various additional processes through which mindfulness may contribute to relationship satisfaction (for a detailed discussion, see Karremans et al. 2017). For example, (the training of) mindfulness has been associated with basic cognitive skills like executive control and emotion regulation that benefit romantic relationship functioning (Chambers et al. 2009; Goldin and Gross 2010; Teper and Inzlicht 2013). Moreover, there is some support that mindfulness promotes access to otherwise implicit negative feelings and emotions (e.g., Brown and Ryan 2003), which may help partners to better regulate those feelings rather than to act upon them in an automatic fashion. Finally, mindfulness has been associated with relationship-enhancing factors like empathy, compassion, and secure attachment (Birnie et al. 2010; Hertz et al. 2015). Future research should further explore how these different processes, separately or in interaction, explain the link between mindfulness and romantic relationship satisfaction. Hopefully, the current findings provide a springboard to study such additional questions.

## Data Availability

Additional online material can be found on the Open Science Framework: osf.io/8apa6/. Data can be obtained from the first author on request.

## Appendix

### Partner Acceptance Scale

The Partner Acceptance Scale is a 5-item scale measuring to what extent the respondent acknowledges his/her partner’s imperfections without feeling the urge to change them. Items 1, 4, and 5 should be reverse coded.

Instructions: In the following you read several statements that describe how people relate to their romantic partner. Please indicate to what degree the statement describes your own stance toward your partner on a scale from 1 (totally disagree) to 7 (totally agree).

**Table:**

	1	2	3	4	5	6	7
1. “My partner does not have to be the perfect partner.”							
2. “I try to change the things which I do not like about my partner.”							
3. “I can accept the less pleasant characteristics of my partner.”							
4. “Frankly, I would like my partner to be the ideal partner. “							
5. “I find it hard to accept that my partner has also less pleasant characteristics.”							
